# Managing Hereditary Angioedema in a Three-Generation Family: Danazol’s Promise in Resource-Limited Settings

**DOI:** 10.7759/cureus.74481

**Published:** 2024-11-26

**Authors:** Pradnya M Joshi, Mohd Saeed Siddiqui, Ajinkya Deshmukh, Madhuri B Engade, Mohammad Haseeb, Rishitha Reddy, Supriya Kubde

**Affiliations:** 1 Department of Pediatrics, Mahatma Gandhi Mission (MGM) Medical College and Hospital, Aurangabad, IND; 2 Department of Internal Medicine, Mahatma Gandhi Mission (MGM) Medical College and Hospital, Aurangabad, IND

**Keywords:** autosomal dominant inheritance, c1 inh, c4 levels, danazol, hereditary angioedema, long term prophylaxis, resource-limited settings, severe attacks

## Abstract

Background

Hereditary angioedema (HAE) is a rare disorder in India, and while prevalence data is limited, it is believed that a significant number of individuals may be affected. Due to restricted access to first-line treatments, older therapies like danazol are commonly used despite associated risks in resource-constrained settings. This study aimed to assess the efficacy of danazol as an affordable long-term prophylaxis (LTP) for HAE in a three-generation family.

Methods

In this retrospective study, we analyzed demographic and clinical data from 22 HAE patients within a three-generation family, assessing serum C4 and C1 esterase inhibitor levels to confirm diagnosis. Patients were treated with danazol for LTP, and the effectiveness of treatment was evaluated using the angioedema control test (AECT), alongside monitoring for adverse effects. A paired-sample t-test was used to compare AECT scores before and after treatment.

Results

The participants had a mean age of 33.5 years and a male predominance (63.63%). The mean age of symptom onset was 20.4 years, and six died due to laryngeal edema episodes. Nearly all patients exhibited low serum C1-INH levels, confirming the diagnosis. Among the nine patients treated with danazol for LTP, AECT scores significantly improved from a mean of 7.9 to 9.8 (p = 0.003), with 90% experiencing reduced attack frequency. Mild adverse effects, such as weight gain and menstrual irregularities, were observed in 44.4% of treated patients.

Conclusions

This retrospective study demonstrated that danazol reduced attack frequency and severity in HAE patients in a resource-limited setting, with mild adverse effects observed in some individuals. While these findings support danazol as a cost-effective option for managing HAE, the results should be interpreted with caution due to the study’s design limitations. The AECT proves to be a useful tool for evaluating the control of angioedema attacks. Because the risk of adverse effects is high, close monitoring of patients is mandatory. However, many patients accept the adverse effects of prophylactic treatment to avoid the distressing and sometimes life-threatening attacks of this condition. Regular monitoring remains essential to mitigate adverse effects, and prospective clinical trials are necessary to further validate the efficacy and safety of danazol for LTP.

## Introduction

Hereditary angioedema (HAE) is an uncommon disorder characterized by recurrent episodes of non-itchy subcutaneous and/or submucosal swellings. The estimated prevalence of HAE ranges from approximately 1 in 10,000 to 1 in 50,000 individuals; however, there are no prevalence data specific to India or other developing nations. Current estimates suggest that between 27,000 and 135,000 individuals may be affected by HAE in India, and many remain undiagnosed [1,2].

Unfortunately, many of these cases remain undiagnosed. The standard treatment for acute episodes of angioedema involves the replacement of plasma-derived or recombinant C1-INH protein administered intravenously for on-demand treatment and short-term prophylaxis (STP) [3]. Until recently, first-line treatment options for on-demand treatment or prophylaxis were not available in India; consequently, physicians resorted to using fresh frozen plasma for both on-demand treatment and STP [4,5]. For long-term prophylaxis (LTP), attenuated androgens (AAs; danazol or stanozolol) and/or tranexamic acid (TXA) were commonly employed [6,7]. While these agents have demonstrated utility for LTP, they are associated with significant adverse effects - particularly AAs - and incomplete efficacy in the case of TXA [2,8,9]. AAs are contraindicated during pregnancy and are not recommended for use in children [3,9]. The lack of access to first-line treatments presents a considerable challenge for patients suffering from HAE in India as well as for their healthcare providers. Although intravenous pd-C1-INH has recently become available in India as a first-line treatment option, the absence of universal health insurance remains a significant barrier to access for both patients and physicians. HAE imposes substantial direct medical costs on patients and their families, along with indirect costs associated with lost productivity [8]. Moreover, diagnostic facilities are often limited in many developing countries; thus, adherence to international evaluation algorithms may not be feasible [1,2].

International guidelines recommend that all HAE attacks be considered for on-demand treatment. Despite being a life-threatening condition, diagnosis is frequently overlooked due to resource limitations. In this study, we identified 22 affected family members who experienced significant restrictions in daily activities due to recurrent HAE attacks. We aimed to evaluate the efficacy of danazol as a more affordable long-term prophylactic treatment option while employing a subjective assessment tool (angioedema control test, AECT) to assess treatment response.

## Materials and methods

Study design and setting

This descriptive, retrospective study was conducted over a period of 18 months at Mahatma Gandhi Mission (MGM) Medical College and Hospital in Aurangabad, India. The study focused on the demographic and clinical data derived from the medical records of 22 patients diagnosed with HAE from a three-generation family. All patients were previously inadequately controlled regarding HAE management.

Study population

The study population included symptomatic patients and affected family members identified through clinical history and family screening. The first index case was identified as a 34-year-old patient (proband) who presented with an acute laryngeal attack. Through detailed family history, 28 other symptomatic members from three generations were identified, and 22 were included in the analysis. Six family members had died from laryngeal edema-related asphyxia prior to the study due to the unavailability of appropriate treatment. Those with a confirmed diagnosis of HAE based on clinical presentation and laboratory investigations were included.

Clinical and laboratory assessment

The diagnosis of HAE was based on clinical features and laboratory confirmation. All symptomatic patients and affected family members underwent serum complement C4 and C1 esterase inhibitor level assessments. C4 complement levels and C1 esterase inhibitor (C1-INH) levels were measured using a method following standard clinical laboratory protocols (C4 levels by immunoturbidimetry and C1inh levels by Nephelometry). The HAE diagnosis was confirmed when both low serum C4 levels and reduced or dysfunctional C1-INH levels were detected.

Treatment protocol

Due to limited access to first-line therapies for HAE in India and financial constraints, LTP with danazol, an AA, was offered to consenting individuals. The initiation dose of danazol ranged from 200 to 400 mg per day, and doses were adjusted based on the patient’s response and tolerance to the medication. Treatment duration varied from two months to one year, depending on patient adherence, response to treatment, and occurrence of adverse effects.

Data collection and outcome measures

Clinical data were extracted from the patient’s medical records, including demographic information (age and sex), age at symptom onset, frequency and type of attacks (e.g., abdominal pain, facial swelling, limb swelling, and laryngeal edema), and the number of attacks before and after starting danazol. Data were collected for the period of 18 months. The severity of attacks was categorized based on clinical presentation, with life-threatening laryngeal edema being the most severe form of attack.

To evaluate treatment efficacy, we used the AECT, a validated patient-reported outcome measure designed specifically for patients with recurrent angioedema. The AECT includes questions about attack frequency, severity, and the impact of angioedema on the patient’s daily life. The AECT score ranges from 0 to 16, with higher scores indicating better disease control. A score of 16 reflects complete control of the condition, while scores below 10 are indicative of poorly controlled angioedema [7]. In this study, treatment efficacy was defined as an improvement in AECT score from baseline, reflecting reduced attack frequency or severity. In one study, the lowest AECT score change that indicates a meaningful improvement in disease control was found to be three points [10].

Adverse effects monitoring

Throughout the treatment period, patients were closely monitored for any adverse effects associated with danazol. Side effects of concern included weight gain, virilization, menstrual irregularities, headache, hypertension, and abdominal discomfort, as reported in previous studies of danazol use. Any serious adverse effects or reasons for discontinuation of treatment were recorded.

Statistical analysis

Data were analyzed using Jamovi software (version 2.4.1) [11]. Descriptive statistics were used to summarize demographic data, attack frequency, and severity. Continuous variables such as AECT scores before and after treatment were compared using paired-sample t-tests to determine the statistical significance of changes observed during danazol therapy. A p-value of <0.05 was considered statistically significant. Additionally, Cohen’s d was calculated to assess the effect size of the intervention on disease control.

Ethical considerations

Ethical approval was obtained from the Institutional Ethical Committee of MGM Medical College and Hospital, Aurangabad. All patients included in the study provided informed consent for the use of their clinical data for research purposes. Confidentiality of patient information was maintained throughout the study, and no identifiable information was disclosed.

## Results

**Figure 1:**
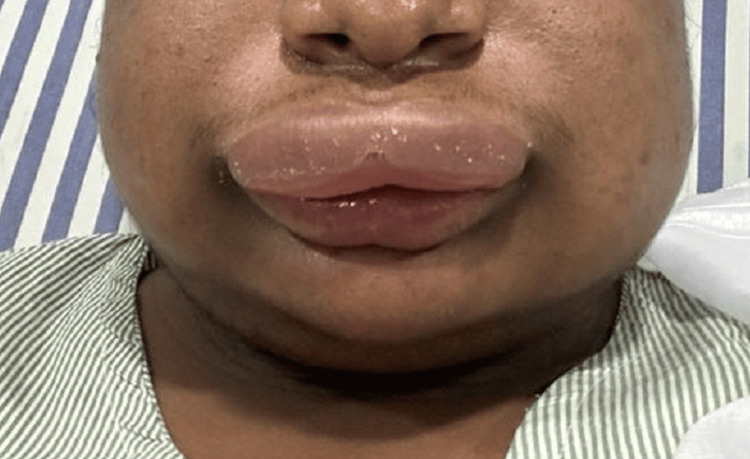
Clinical photograph of proband during acute attack of HAE HAE, hereditary angioedema

The study involved 22 symptomatic HAE patients from a three-generation family. The initial symptomatic case, or proband, was a 34-year-old male who presented with an acute laryngeal attack, prompting further investigation into his family history (Figure 1).

**Figure 2:**
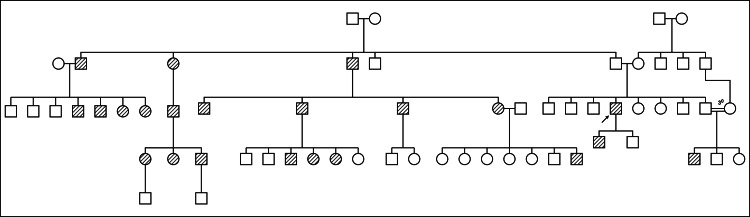
Three-generation pedigree chart of the family The arrow indicates the proband.

Through this historical inquiry, 21 additional symptomatic family members were identified (Figure 2), all of whom displayed recurrent episodes consistent with HAE.

The mean age of the study cohort was 33.5 years, with a male predominance, as 63.63% (14/22) of the patients were male. The average age at symptom onset was 20.4 years (range: 16-24 years). Patients reported experiencing biweekly attacks, with an average frequency of two to three episodes per fortnight. The clinical presentation of these attacks varied, involving abdominal pain, facial swelling, and limb swelling, alongside life-threatening laryngeal edema. Sadly, six family members had died from asphyxiation due to untreated laryngeal edema, underscoring the severity of this condition and the critical need for timely intervention.

Laboratory investigations revealed that nearly all patients exhibited deficiencies in serum complement C4 and C1 esterase inhibitor (C1-INH) levels, confirming the diagnosis of HAE. The mean serum C1-INH level across the cohort was recorded at 0.1 mg/dL, significantly below the normal range of 0.21-0.39 mg/dL (Table 1).

**Table 1:** Characteristics of the study participants

Characteristics	Overall (N = 22)
Age	
Mean (SD)	33.5 (18.1)
Range	5.0-65.0
Gender	
F	8 (36.36%)
M	14 (63.63%)
Age of symptom onset	
N-Miss	4
Mean (SD)	20.4 (5.4)
Range	12.0-35.0
Serum C1 Inh levels (N: 0.21-0.39)	
N-Miss	3
Mean (SD)	0.1 (0.1)
Range	0.0-0.4
Danazol duration (months)	
Mean (SD)	1.5 (3.0)
Range	0.0-12

Out of the 22 symptomatic patients, nine individuals who consented to this treatment, as recorded in their medical records, were initiated on LTP with danazol due to financial constraints that limited access to other treatments was documented as part of the clinical management for patients. Danazol was administered over a variable duration of two months to one year, and it demonstrated significant clinical efficacy. Due to limited access to first-line therapies for HAE in India and financial constraints, LTP with danazol, an AA.

For the 13 symptomatic patients who declined danazol treatment, detailed data on their disease progression or alternative management approaches were not consistently documented, as some patients did not continue regular follow-up. This limitation precluded a comparative analysis between treated and untreated groups.

**Figure 3:**
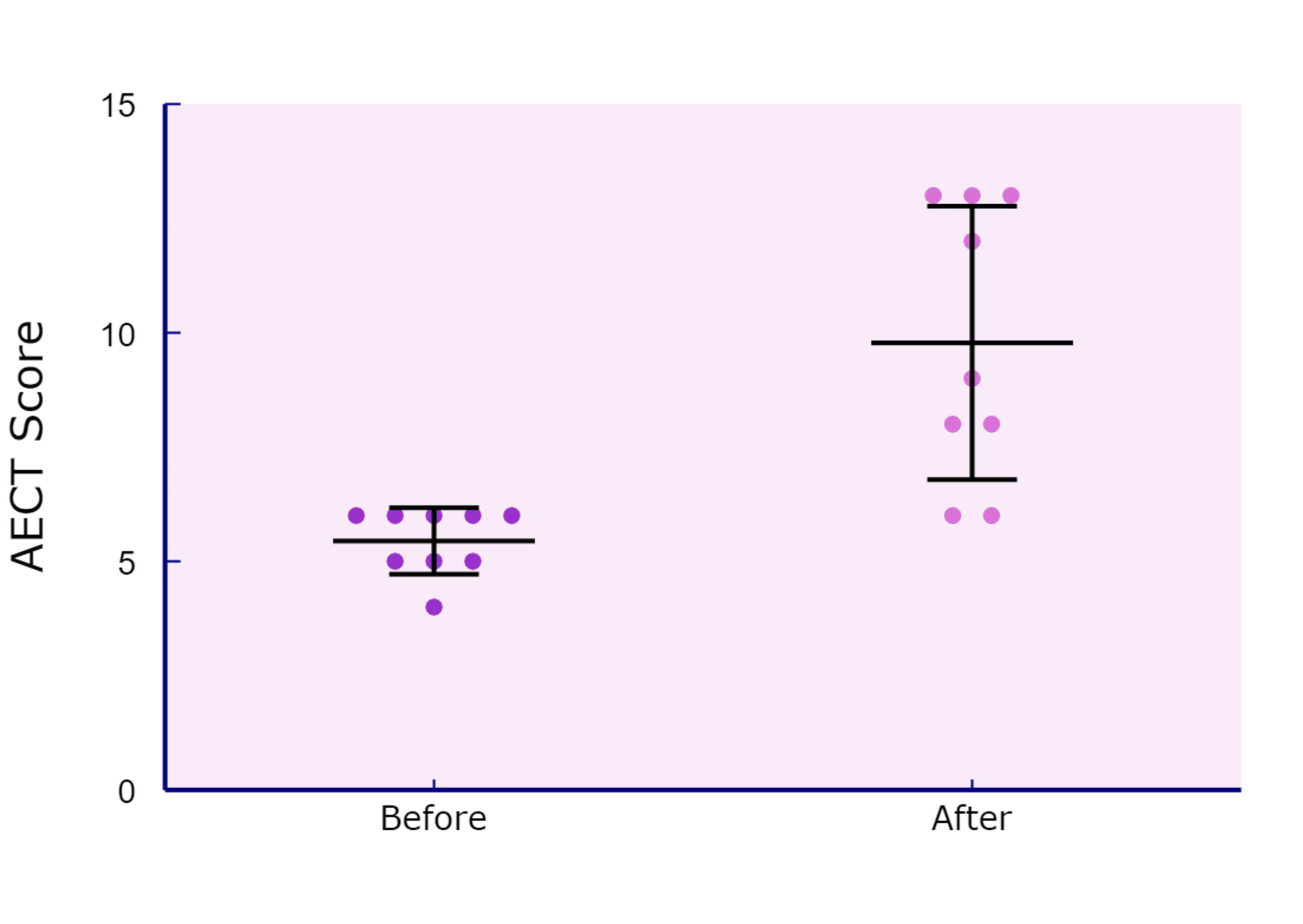
AECT score before and after LTP with danazol in study participants AECT, angioedema control test; LTP, long-term prophylaxis

A comparison of AECT scores before and after danazol treatment revealed a statistically significant improvement. Pre-treatment AECT scores had a mean of 7.9 (SD = 3.3), while post-treatment scores improved to a mean of 9.8 (SD = 3.0). A paired-sample t-test confirmed that this increase in AECT scores was statistically significant, t (8) = -3.79, p = 0.003, with a large effect size (Cohen’s d = -1.26), indicating that the improvement was not only statistically significant but also clinically meaningful (Figure 3).

Despite the overall efficacy of danazol, mild adverse effects were reported in 44.4% (4/9) of the treated patients. The most reported side effects included weight gain, virilization (manifested as menstrual irregularities), headaches, and mild abdominal discomfort. These adverse effects were generally well tolerated, and none of the patients discontinued therapy due to these issues.

## Discussion

In the past decade, significant advancements have been made in the treatment of HAE, including new drugs and expanded uses for existing treatments. Increased understanding of the pathophysiology of C1-INH-HAE, particularly the role of the kallikrein-kinin system, has facilitated the development of novel therapies. Inhibition of plasma kallikrein and activated factor XII has been central to this progress, with several new treatments now available for acute angioedema attacks, including C1-INH concentrate, icatibant acetate, ecallantide, and recombinant C1-INH. These advancements have transformed the management of HAE, particularly in developed settings. The four drugs that are currently available for the treatment of acute angioedema attacks (purified plasma-derived human C1 esterase inhibitor concentrate, icatibant acetate, ecallantide, and recombinant human C1 esterase inhibitor) are all authorized for self-administration, except ecallantide [12].

The management of HAE involves three main therapeutic strategies: treatment of acute attacks, STP, and LTP. When determining LTP, individual factors such as efficacy, safety, mode of administration, frequency, and preexisting comorbidities need to be considered. Studies have shown that shared decision-making between patients and physicians, taking patient preferences into account, improves adherence to long-term therapy and outcomes [13].

Our findings align with previous studies on the efficacy of danazol in managing HAE. Bork et al.’s study [14] demonstrated that danazol significantly reduced the frequency and severity of attacks, with nearly 50% of patients becoming symptom-free. Similarly, in our study, danazol provided a substantial clinical benefit, as indicated by improvements in the AECT scores, with 90% of patients experiencing reduced attack frequency.

Since its introduction in the 1980s, danazol has been a mainstay of HAE prophylaxis, particularly in resource-limited settings like China and India, where more expensive alternatives may not be accessible. In a study conducted in China [15], danazol showed satisfactory efficacy, with 96% of patients achieving complete control of their symptoms at higher doses. Our study similarly found that danazol remains a valuable option in resource-constrained environments where access to newer therapies is limited.

Danazol has the added advantage of oral administration and low cost. The mechanism of danazol in the treatment of HAE is unclear, but it may enhance the synthesis of C1-INH in monocytes and hepatoma cell lines. Although there is evidence for the efficacy of danazol in C1-INH-HAE, there have been no reports on its application status for the treatment of C1-INH-HAE in India.

Gelfand et al. [16] conducted a double-blind placebo-controlled trial with danazol and concluded that it effectively prevents attacks in HAE and acts to correct the underlying biochemical abnormality (i.e., C1 esterase inhibitor levels increased three to four times, and levels of the fourth component of complement (C4) increased 15 times).

A survey conducted by Troelnikov et al. [17] in South Australia in 2024 indicated that current LTP therapies, including danazol, lanadelumab, and C1-inhibitor, applied to appropriate patients taking into account disease activity and drug risks and tolerance, are effective for HAE attack prevention and produce high levels of satisfaction.

Our study found some or good benefits in almost all patients with HAE taking prophylaxis with Danazol (improvement was assessed with the AECT). While danazol effectively prevents attacks, its long-term use is associated with potential side effects. In our study, 44.4% of patients experienced mild adverse effects, including weight gain, virilization, and headaches. These findings are consistent with the case series by Maurer et al. [18], which reported similar adverse effects among HAE patients taking danazol. Given these risks, regular monitoring of liver function, blood pressure, hematocrit, creatine kinase level, and lipid levels is recommended during long-term danazol therapy. Also, liver ultrasonography should be performed once a year. However, this was challenging in our study due to patient unwillingness and affordability issues, an issue that is often encountered in resource-limited settings.

Throughout the treatment period, adverse effects associated with danazol were documented in patient medical records, as recorded by the treating physicians. Side effects of concern included weight gain, virilization, menstrual irregularities, headache, hypertension, and abdominal discomfort, consistent with adverse effects reported in previous studies of danazol use. However, it is acknowledged that other concurrent medications or treatments, if used, were not systematically documented, making it challenging to attribute adverse effects solely to danazol. Adverse effects monitoring was conducted during routine follow-up visits, but the frequency and consistency of these assessments were influenced by resource limitations and patient adherence to follow-up schedules. Serious adverse effects or reasons for discontinuation of treatment, where documented, were included in the analysis. The study’s generalizability is limited by its small sample size and focus on a single family. One limitation of our study is the retrospective design, which prevents a comprehensive assessment of danazol discontinuation and its impact on attack recurrence. Information on gynecological history, including prior use of danazol or similar agents for unrelated conditions, was not consistently available in the records and was therefore not analyzed in this study.

A key limitation of this retrospective study is the inability to account for all potential confounding factors, such as the use of other treatments or interventions during the study period. While we did not specifically document concurrent therapies, it is possible that some patients may have used additional medications or supportive treatments, which could have influenced their attack frequency and severity. This highlights the inherent challenges of retrospective designs, where data completeness relies heavily on available records. Future prospective studies are essential to control for these confounding factors and provide a clearer understanding of the isolated effects of danazol. Additionally, the consistency of AECT administration and adverse effects monitoring was influenced by variability in patient comprehension and literacy levels, which may have affected the accuracy of self-reported data. The literature suggests that discontinuing AAs often leads to an increase in HAE attack frequency and severity [19]. This area remains underexplored in our patient cohort due to follow-up challenges. Retrospective studies are often chosen for rare diseases like HAE because prospective studies face difficulties such as limited patient recruitment, short observation periods, and the potential for patients to be lost to follow-up. Additionally, patients may use other medications that could confound the results. A comparative analysis of danazol with alternative LTP options like TXA could provide valuable insights into their relative efficacy, safety, and cost-effectiveness, particularly in resource-limited settings. Future studies should explore such comparisons to better inform clinical decision-making and optimize long-term outcomes for HAE patients.

In a nutshell, our study suggests that danazol can be an effective long-term prophylactic treatment for HAE in resource-limited settings. Its affordability and oral administration make it a viable option where access to newer, more expensive therapies is limited. However, the lack of follow-up post-treatment discontinuation, reliance on AECT as the sole measure of efficacy, and absence of data on quality of life (QoL) or functional status represent significant limitations in assessing the full impact of danazol in this cohort. These factors underscore the need for prospective studies that incorporate objective outcome measures, long-term follow-up, and comprehensive assessments of QoL to better evaluate the efficacy and safety of danazol.

## Conclusions

This retrospective study demonstrated that danazol reduced attack frequency and severity in HAE patients in a resource-limited setting, with mild adverse effects observed in some individuals. While these findings support danazol as a cost-effective option for managing HAE, the results should be interpreted with caution due to the study’s design limitations. The AECT proves to be a useful tool for evaluating the control of angioedema attacks. Because the risk of adverse effects is high, close monitoring of patients is mandatory. However, many patients accept the adverse effects of prophylactic treatment to avoid the distressing and sometimes life-threatening attacks of this condition. Regular monitoring remains essential to mitigate adverse effects, and prospective clinical trials are necessary to further validate the efficacy and safety of danazol for LTP.
